# Endothelial lesion and complement activation in patients with Scleroderma Renal Crisis

**DOI:** 10.1590/2175-8239-JBN-2018-0202

**Published:** 2019-02-25

**Authors:** Ney Arencibia Pérez, María Luisa Agüera Morales, Rafael Sánchez Sánchez, Rosa María Ortega Salas, Rafael Ángel Fernández de la Puebla, Mario Espinosa Hernández

**Affiliations:** 1 Reina Sofia University Hospital, Nephrology, Av. Menendez Pidal, s/n Córdoba, Spain.; 2 Reina Sofia University Hospital, Pathological Anatomy, Cordoba, Spain.; 3 Reina Sofia University Hospital, Internal Medicine, Cordoba, Spain.

**Keywords:** Scleroderma, Localized, Thrombotic Microangiopathy, Systemic Sclerosis, Hypertensive Retinopathy, Endothelium, Complement Activation, Esclerodermia Localizada, Microangiopatia trombótica, Esclerose Sistêmica, Retinopatia Hipertensiva, Endotélio, Ativação do Complemento

## Abstract

In kidney biopsies reviews, scleroderma renal crisis (SRC) is characterized by vascular endothelial injuries, C4d deposits on peritubular vessels, and acute and chronic injuries coexisting on the same biopsy. The clinical signs of thrombotic microangiopathy (TMA) are described in systemic sclerosis (SSc), nevertheless, it has not been related to acute injuries described on kidney biopsies. We report a case of SRC in a patient with scleroderma-dermatomyositis overlap syndrome, which also showed clinical and histopathological data of TMA. On fundus examination, a severe acute hypertensive retinopathy was found. The kidney biopsy showed severe endothelial damage with widening of mucoid cells at the level of the intima, focal concentric proliferation on most small arterioles, and C3, C4d, and IgM deposits along the capillary walls. The genetic study of complement only showed the presence of membrane cofactor protein (MCP) risk haplotypes, without other genetic complement disorders. We understand that in a patient with TMA and SSc, the kidney damage would be fundamentally endothelial and of an acute type; moreover, we would observe clear evidence of complement activation. Once further studies correlate clinical-analytical data with anatomopathological studies, it is likely that we will be forced to redefine the SRC concept, focusing on the relationship between acute endothelial damage and complement activation.

## Resumo

Nas revisões de biópsias renais, a crise renal esclerodérmica (CRE) é caracterizada por lesões endoteliais vasculares, depósitos de C4d em vasos peritubulares e lesões agudas e crônicas que coexistem na mesma biópsia. Os sinais clínicos de microangiopatia trombótica (MAT) são descritos na esclerose sistêmica (ES); no entanto, não foram relacionados às lesões agudas descritas nas biópsias renais. Relatamos um caso de CRE em um paciente com síndrome de superposição de esclerodermia-dermatomiosite, que também apresentou dados clínicos e histopatológicos de MAT. No exame de fundo do olho, foi encontrada uma retinopatia hipertensiva aguda grave. A biópsia renal mostrou lesão endotelial grave com alargamento das células mucoides ao nível da íntima, proliferação concêntrica focal na maioria das pequenas arteríolas e depósitos de C3, C4d e IgM ao longo das paredes dos capilares. O estudo genético do complemento mostrou apenas a presença de haplótipos de risco da proteína cofator de membrana (PCM), sem outros distúrbios genéticos do complemento. Entendemos que em um paciente com MAT e ES, o dano renal seria fundamentalmente endotelial e do tipo agudo; além disso, observaríamos evidências claras de ativação do complemento. Uma vez que novos estudos correlacionam dados clínico-analíticos com estudos anatomopatológicos, é provável que sejamos forçados a redefinir o conceito de CRE, enfocando a relação entre dano endotelial agudo e ativação do complemento.

## Introduction

Classically, scleroderma renal crisis (SRC) is defined as the association of acute renal injury, normal urinalysis or mild proteinuria, and hypertension (often malignant hypertension), typically associated with plasma renin increase^1^^,^ 2 ^,^ 3.

SRC is an uncommon complication but between 20 and 30 percent of patients do not recover renal function^4^^,^ 5 ^,^ 6. Although it is a severe disease, to this day there are only a few histological descriptions of kidney biopsies.

In kidney biopsies reviews, SRC is characterized by vascular endothelial injuries, C4d deposits on peritubular vessels, and acute and chronic injuries coexisting on the same biopsy^6^. The clinical signs of thrombotic microangiopathy (TMA) are described in systemic sclerosis (SSc); nevertheless, TMA has not been related to acute injuries described on kidney biopsies.

We present a clinical case with SRC, including the anatomopathological observations and clinical findings, and demonstrate the role of complement activation.

## Case report

A 50-year-old man was transferred to the emergency unit of our hospital due to progressive loss of vision in the previous 5 days. He had a 2-year history of Raynaud syndrome treated with pentoxifylline 600 mg/24 h. Two weeks prior to admission, his rheumatologist suspected of a dermatomyositis and scleroderma overlap syndrome because the patient started with dysphagia and constitutional syndrome, and previously, he had Gottron nodules.

On arrival at the emergency unit, his blood pressure was 220/120 mmHg. Laboratory studies showed creatinine (Cr) of 1.7 mg/dL (two months before, Cr was 0.9 mg/dL) associated with a lactate dehydrogenase (LDH) of 1252 U/L, creatine phosphokinase (CPK) of 3984 U/L, hemoglobin of 10.9 g/dL, platelet count of 149,000/uL (two months before was 326,000/uL), schistocytes at 2.5% and decreased levels of haptoglobin (13 mg/dL). Uroanalysis showed protein/Cr ratio of 0.3 and microscopic hematuria with 13 red blood cells/mL. Renal ultrasonography and Doppler study were normal.

We were getting results from the blood tests during his hospitalization. ANA test was positive (1/640) but anti-dsDNA was negative, C3 and C4 were depressed (C3 was 65.6 mg/dL and C4 was 11.5 mg/dL), anticentromere, ANCA-MPO, ANCA-PR3 and anti-GBM antibodies were negative. Protein electrophoresis with immunofixation and quantification of serum immunoglobulins (IgG, IgM and IgA) were normal, as well as blood levels of vitamin B12 and folic acid. Tests for HIV, hepatitis C virus, and hepatitis B virus were negative.

**Figure 1:**
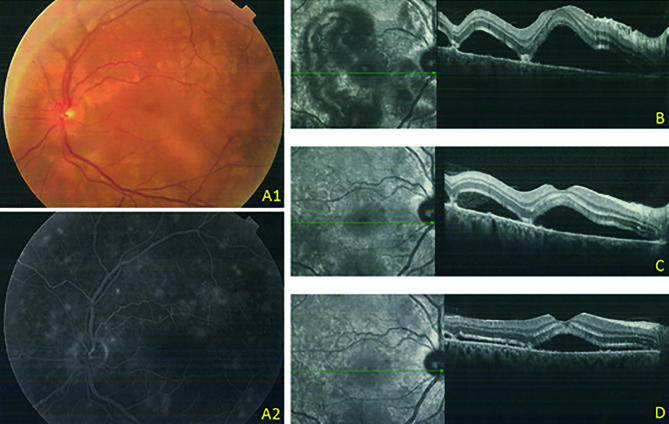
A1. Dilated fundus examination showing multi-foci serous retinal detachment. A2: Fluorescent angiography showing multiple hypofluorescent foci on the posterior pole. B: Macular optical coherence tomography at the 1st day of admission, C, 3rd day of admission, and D, 10th day of admission (progressive improvement of retinal detachment is observed).

Regarding severe hypertension, fundus examination showed a severe acute hypertensive retinopathy characterized by papillary edema with serous multifocal retinal detachments on the posterior pole of both eyes (Figure 1). Plasma renin level was increased to 320.1 pg/mL (1.8-59.4). Plasma cortisol, 24-hour urine fractionated catecholamines, and plasma aldosterone were normal.

Due to increased CPK, Raynaud syndrome, scapular waist muscle weakness, and anti-Ro/SSA and anti-PM-scl-75/PM-scl-100 positivity, a nailfold capillaroscopy and electromyographic study were performed.

Nailfold capillaroscopy demonstrated significant dropout (loss of capillary loops), lower density, and dilatation and tortuosity of capillaries, which are related to dermatomyositis. Electromyogram showed a pattern of diffuse myopathic involvement of inflammatory features. With these findings, and the Gottron nodules, the diagnosis of scleroderma-dermatomyositis overlap syndrome was confirmed^7^^,^ 8 ^,^ 9.

Within the context of this disease, associated with acute renal injury and microangiopathic hemolytic anemia, a renal biopsy was performed. Direct coombs test was negative and blood activity of ADAMTS13 was normal.

**Figure 2:**
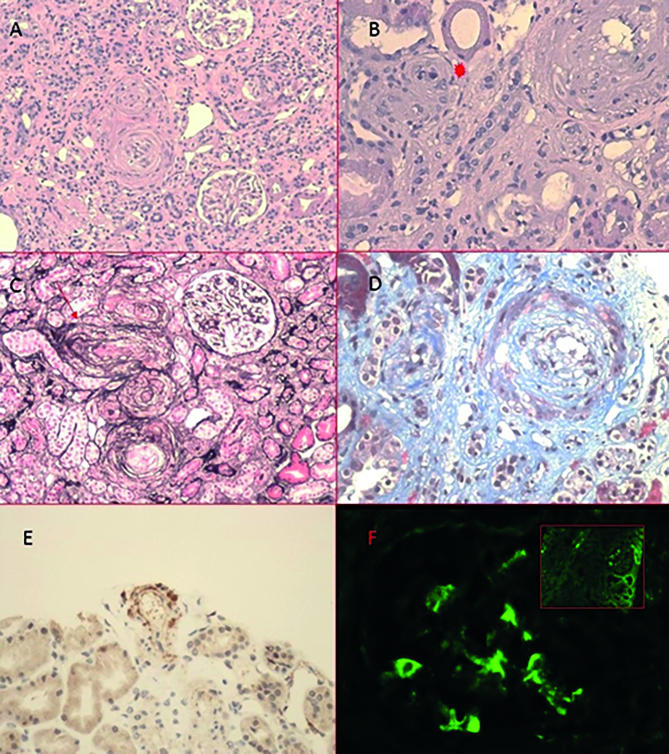
A (100×) and B (200×): HE staining of arterioles with intimal proliferation and thickening that leads to obliteration of the vascular lumen and degraded red blood cells (image B, red star). C: Red arrow indicating arteriole with concentric "onion-skin" hypertrophy (methenamine silver stain, 100×). D: Arterioles with mucoid intimal thickening (Masson trichrome, 200×). E: Granular and focal immunostaining of small caliber artery (C4d, 200×). F: C3 and IgM (top right) immunodeposits along capillary walls and in te mesangium.

The histopathology report stated a severe endothelial damage with widening of mucoid cells at the level of the intima, slight tubular atrophy, and focal concentric proliferation on most small arteries and arterioles. On direct immunofluorescence (IF) there was C3 and IgM deposits along the capillary walls in the mesangium and small vessels. Immunohistochemistry was positive for C4d on small-caliber arteries (Figure 2).

Regarding the genetic-molecular complement study, we observed low serum C3 and C4 levels, indicating an activation of the classical and alternative complement pathways. Antibodies to complement factor H were negative, and serum factor H, membrane cofactor protein (MCP), and FI levels were normal. The genetic study only showed the presence of MCP risk haplotypes, without other genetic complement disorders.

Three months after admission, after controlling for blood pressure with angiotensin-converting enzyme (ACE) inhibitors, the patient continued with asthenia but without evidence of hemolytic anemia and renal failure (Cr 0.8 mg/dL without proteinuria and hematuria.). Furthermore, he recovered from vision loss and muscle weakness^10^^,^ 11.

## Discussion

Our patient had renal failure associated with secondary TMA due to SSc overlapped with a dermatomyositis. In general, renal replacement therapy (RRT) is required in approximately 50% of patients with secondary TMA and acute renal injury^12^. In cases of secondary TMA due to SSc, RRT is required in 20-25% of cases^4^^,^ 5 ^,^ 6. In this patient, early diagnosis and management provided a complete recovery of renal function and other systemic manifestations.

Until now, kidney biopsies are not routinely indicated for all SRC. However, when SRCs are associated to other systemic diseases, kidney biopsy is particularly recommended^3^, and takes on even greater importance to assess the levels of chronicity and endothelial injury, allowing the estimation of the reversibility degree. In our patient, the kidney biopsy took on greater relevance because it was obtained at early stages of the disease, with only few mild chronic injuries.

Although several reviews have reported that endothelial damage is the underlying problem in TMA, in the case of SSc, the physiopathogenic mechanisms that produce endothelial damage are unclear. In early stages, a SRC may produce an acute endothelial damage and with time, give rise to mucopolysaccharide deposits and myointimal proliferation or fibrinoid necrosis due to a persistent vascular remodeling, establishing chronic endothelial damage. The result is a retraction and necrosis of the glomeruli^3^.

In our patient, we uphold that there was a direct association between endothelial injury and complement activation (classical and alternative complement pathways). We observed low plasma C3 and C4 levels, and C3 and C4d deposits on dIF and immunohistochemistry respectively, suggesting a systemic TMA with complement activation^13^.

In the complement genetic study, we did not identify any mutation in the regulatory genes, however, the patient had risk haplotypes in MCP, of which, to this day, the pathogenic role is unknown.

The patient was treated early with ACE inhibitors, prior to the establishment of chronic kidney damage^3^^,^ 4 ^,^ 5 ^,^ 6. The initial therapy with eculizumab in SRC with TMA could be an alternative, but the available data are based on small case series. Results from a Spanish study showed that short treatments with eculizumab were beneficial in patients with secondary TMA, but two cases with SRC did not respond to treatment^12^.

In summary, this is a clear case of SRC with clinical and histopathological data of acute endothelial damage and complement activation. TMA in a patient with SSc would lead to kidney damage of an endothelial and acute type; moreover, we would observe clear evidence of complement activation (C4d positive on the vascular wall, C3 on dIF, and low plasma levels of C3 and C4). However, this needs to be corroborated by case series and experimental evidence and then, once we correlate clinical-analytical data with anatomopathological studies, it is likely that we will be forced to redefine the SRC concept, focusing on the relationship between acute endothelial damage and complement activation.
